# Pharmacovigilance practice among pediatric neurologists from Poland and Germany

**DOI:** 10.1186/s12909-023-04542-4

**Published:** 2023-08-01

**Authors:** Dorota Kopciuch, Krzysztof Kus, Izabela Niśkiewicz, Jędrzej Fliciński, Tomasz Zaprutko, Piotr Ratajczak, Elżbieta Nowakowska, Karolina Hoffmann, Agnieszka Koligat-Seitz, Wiesław Bryl, Anna Paczkowska

**Affiliations:** 1 Department of Pharmacoeconomics and Social Pharmacy, Poznan University of Medical Sciences, Rokietnicka 7 St, 60-806 Poznań, Poland; 2 Department of Neurology, Martin-Luther-University Hospital Halle- Wittenberg, Halle, Germany; 3 Department of Developmental Neurology, Poznan University of Medical Sciences, Poznan, Poland; 4 Department of Pharmacology and Toxicology Institute of Health Sciences, Collegium Medicum, University of Zielona Gora, Góra, Poland; 5 Department of Internal Diseases, Metabolic Disorders and Arterial Hypertension, Poznan University of Medical Sciences, Poznan, Poland; 6 Department of Otolaryngology, Greater Poland Pediatric Center, Poznan, Poland

**Keywords:** Pharmacovigilance, Epilepsy, Children with epilepsy, Adverse drug reactions, Drug safety, Neurologists’ knowledge, Poland, Germany

## Abstract

**Objectives:**

To compare the pediatric neurologists’ knowledge, practice, and barriers to the pharmacovigilance (PV) process in Poland and Germany.

**Methods:**

The research tool was an online anonymous questionnaire on Google Forms e-mailed to pediatric neurologists from Poland and Germany.

**Results:**

The questionnaires were handed out to 830 pediatric neurologists and 371 expressed their consent to participate in the study. Most of the neurologists were familiar with the definition of PV and adverse drug reactions (ADRs). Only 34.10% of pediatric neurologists from Poland, and 38.88% from Germany believe that many ADRs are preventable and almost most of them believe it is necessary to report ADRs from children with epilepsy. Unfortunately, in opposite to this knowledge, only 37.79% of respondents from Poland and 40.32% from Germany felt co-responsible for reporting ADRs. The main reason for the neurologists not to report ADRs was a conviction that reporting ADRs would be an additional burden generating extra work.

**Conclusion:**

There is no big difference between the practice of PV by pediatric neurologists in Poland and Germany. System-regulated PV stabilization in the country translates into the practice of maintaining PV. Monitoring the safety of pharmacotherapy and knowledge of risks associated with ADRs should be included in the curricula of academic neurologics courses.

**Supplementary Information:**

The online version contains supplementary material available at 10.1186/s12909-023-04542-4.

## Key points

1. In the case of children with epilepsy, neurologists are those instances who are first informed about alarming symptoms by their patients, therefore full participation and engagement of neurologists in the pharmacovigilance (PV) process in epileptic children are crucial to ensure their safe pharmacotherapy.
2. Most of the neurologists were familiar with the definition of PV and adverse drug reactions (ADRs) and believe it is necessary to report ADRs from children with epilepsy.
3. Only 37.79% of pediatric neurologists from Poland and 40.32% from Germany felt co-responsible for reporting ADRs.
4. The main reason for the neurologists not to report ADRs was a conviction that reporting ADRs would be an additional burden generating extra work.

**Supplementary Information:**

The online version contains supplementary material available at 10.1186/s12909-023-04542-4.

## Plain Language Summary

Epilepsy is a chronic disorder characterized by episodic, gratuitous seizures. Most children with epilepsy (CWE) rely on antiepileptic drugs causing adverse drug reactions (ADRs). Many ADRs are preventable if physicians actively participate in pharmacovigilance (PV), which its pivotal role is to ensure the safety of pharmacotherapy by e.g. permanent control of ADRs. The study aimed to compare the pediatric neurologists’ (PN) knowledge, practice, and barriers to the PV process in Poland and Germany. The research tool was an online anonymous questionnaire on Google Forms e-mailed to PN from Poland and Germany. Only 34.10% of PN from Poland and 38.88% from Germany believe that many ADRs are preventable and almost most of them believe it is necessary to report ADRs from CWE. Unfortunately, in opposite to this knowledge, only 37.79% of respondents from Poland and 40.32% from Germany felt co-responsible for reporting ADRs. The main reason for the neurologists not to report ADRs was a conviction that reporting ADRs would be an additional burden generating extra work. There is no big difference between the practice of PV by PN in Poland and Germany. System-regulated PV stabilization in the country translates into the practice of maintaining PV.

**Supplementary Information:**

The online version contains supplementary material available at 10.1186/s12909-023-04542-4.

## Introduction

Epilepsy is a chronic disorder characterized by episodic, gratuitous seizures. Most people with epilepsy rely on medical treatment with antiepileptic drugs (AEDs) to achieve control of their seizures [1].

The overall aim in the treatment of epilepsy should be complete control of seizures and no adverse drug reaction (ADR) which is defined “as a response to a drug that is noxious and unintended and occurs at doses normally used in man for prophylaxis, diagnosis or therapy of disease, or for modification of physiological function” [2]. ADR is an event, the etiology of which is well-known in many cases, but the identification of its cause is often very challenging, due to factors such as polypharmacy, individual patient factors, or other unspecified causes. This condition leads to significant global financial burdens for both society and the healthcare system. It accounts for approximately 5–20% of hospitalizations worldwide. Therefore, a well-established drug safety surveillance system plays a crucial role in addressing this situation [2–5].

Pharmacovigilance (PV) and monitoring of adverse events help assess the effectiveness and risk of medications, ensuring safe treatment for patients [6–8].

Poland and Germany seem to be countries with a well-founded position of PV. Germany has been a member of the WHO International Program for Drug Monitoring already since 1968, Poland- since 1972, and the National Office for Registration of Medicinal Products receives tens of thousands of ADR reports annually [9].

The current German and Polish PV approach is mostly harmonized within the European Union and International Council for Harmonisation of Technical Requirements for Pharmaceuticals for Human Use (ICH) framework although there are German-specific requirements for company personnel and the handling of Direct Healthcare Professional Communications (DHPCs). The European lifecycle approach, based on ICH, is fthe ollowed and the safety of a medicinal product is assessed continuously throughout the life of the product.

It is assumed that apart from the condition of PV situation in a given health care system, effectively functioning PV depends on many other factors, such as cultural differences in attitudes towards ADRs reporting, in particular, the knowledge of and physicians’ attitude towards PV and ADRs [10].

In the case of children with epilepsy, pediatric neurologists are those instances who are first informed about alarming symptoms by their patients, therefore full participation and engagement of pediatric neurologists in the PV process in epileptic children are crucial to ensure their safe pharmacotherapy.

Our study aims to compare the pediatric neurologists’ knowledge, practice, attitude, and barriers to PVe process among children with epilepsy in Poland and Germany.

### Methodology

This was an international study based on scientific collaboration between Universities in Poland and Germany. The research tool was an online anonymous questionnaire e-mailed to pediatric neurologists. In the case of both countries, we have utilized the services of companies that offer internet search engines developed based on central registries of physicians. The physicians were randomly selested from the list. Informed consent was obtained from participants via online platform. A literature review was conducted before designing the questionnaire. Important questions and topics from the literature were either modified or directly included as items in our questionnaire. The questionnaire contains 19 items (Supplementary materials). Online questionnaire items covered the following: (1) characteristics of the study population, (2) knowledge of pharmacovigilance and adverse drug reactions (3) pharmacovigilance practice, (4) attitude to and (5) barriers to pharmacovigilance for children with epilepsy, and also (6) activities to improve spontaneous ADR reporting.

Neurologists who did not complete the questionnaire within 3 weeks from the initial mailing were contacted a second time by e-mail. After the reminder, the questionnaire was e-mailed a second time to any remaining non-responders [11].

The study was conducted between OCT 2021 and MAR 2023. Statistical analysis was performed using STATISTICA PL 10.0 (StatSoft). The figures were expressed as the mean, SD, max, and min values. The data distribution pattern was not normal (unlike the Gaussian function). Significant differences between % of group results were determined by the analysis of the Test for Proportions.

## Results

The questionnaires were handed out to 830 pediatric neurologists and 371 (213 from Poland, 158 from Germany) expressed their consent to participate in the study, by sending their responses. The response rate was 45%. The age of the neurologists ranged from 39 to 65 years (mean 41.95 years). Duration of the pediatric neurologists’ practice ranged from 3 to 36 years (mean 14.10 years). Most of the pediatric neurologists work at private practice (42.51%) and hospital (33.84%) (Table 1). The average number of children with epilepsy per day was 11.39 (Table 1).

**Table 1:** Demographic characteristics of pediatric neurologists (*n* = 371)

Parameter	PL/DE	General
Age [years; mean (SD)]	40.09 (25.01)/51(31.10)	41.95 (18.32)
Sex [female; N (%)]	215.18(58)/155.82(42)	371(100)
Years of practice [years; mean (SD)]	13.67 (8.01/15.09 (12.05)	14.10 (9.12)
Place of employment; %		
* universities*	16.01/17.77	12.77(6.34)
* hospital*	36.12/31.14	33.84(12.98)
* private practice/private office*	40.23/37.01	42.51(16.01)
* other*	07.64/14.08	10.88(6.04)
children with epilepsy per day [mean (SD)]	21.34(10.11)/17.87(11.09)	11.39(8.12)

Most of the pediatric neurologists were familiar with the definition of pharmacovigilance (PV) (PL-46.90%; DE-55.98%), the purpose of PV (PL-43.78%; DE- 53.19%) and also the definition of ADRs (PL- 62.09%; DE-64.12%) and the purpose of ADRs (PL-66.66%; DE- 59.09%) (Table 2). Knowledge of the above issues mostly was correlated with age (p < 0.05), years of professional experience (p˂0.05) and place of employment (p˂0.05) i.e. younger pediatric neurologists with shorter professional experience and those who work at universities had a better knowledge of the above-mentioned definitions (Table 2).

**Table 2:** Assessment of pediatric neurologists’ knowledge, attitude and practice towards pharmacovigilance and adverse drug reactions (*n* = 371)

	Response % PL/DE	Country/*P* value	Age (%)				Years of experience (%)			Average patient with epilepsy per day(%)		Place of employment (%)			
	57.41/42.58		≤ 30	31–40	41–50	>50	≤ 10	11–20	>20	< 20	≥ 20	universities	hospital	private practice/private office	other
**KNOWLEDGE**															
The most appropriate definition of pharmacovigilance: Correct answer	46.90/55.98	PL	46.01*	31.23	14.32	8.44	48.12^c^	33.89	17.99	42.88	57.12	51.09^	35.03	8.37	5.51
		DE	44.34^a^	36.76	14.16	4.74	53.76^c^	29.09	17.15	60.77^f^	39.23	50.08#	26.77	18.09	5.06
		Pvalue:	NS	NS	NS	NS	NS	0.0515	NS	0.0017	0.0018	NS	NS	0.0002	NS
The most appropriate purpose of pharmacovigilance: correct answer	43.78/53.19	PL	31.07*	34.12	29.00	5.81	40.56	28.44	31.00	58.09	41.91	47.12^	29.04	5.12	18.72
		DE	45.09^a^	28.12	5.6565	21.14	34.10	39.32	26.58	55.90	44.10	27.12	31.87#	33.76	7.25
		Pvalue	0.0127	NS	< 0.0001	< 0.0001	0.0384	0.0263	NS	NS	NS	< 0.0001	0.0143	< 0.0001	0.0002
Definition of ADR: correct answer	62.09/64.12	PL	39.10*	35.43	17.85	7.62	39.76	30.44	29.80	43.43	56.574	29.97	34.12#	30.10	5.81
		DE	22.65	29.13	19.95	28.27	33.10	37.32	29.58	57.77	42.23	40.09^	28.12	10.65	21.14
		Pvalue	< 0.0001	NS	NS	< 0.0001	NS	NS	NS	0.0006	NS	0,0112	NS	< 0.0001	< 0.0001
The purpose of an ADRs: correct answer	66.66/59.09	PL	43.45^a^	31.96	6.97	17.62	38.46	36.12	25.42	28.99^f^	71.01	42.97	22.12^	31.10	3.81
		DE	40.34*	31.92	17.76	9.98	48.87^c^	33.32	17.81	43.71	56.29	40.09^	28.12	10.65	21.14
		Pvalue	NS	NS	NS	0.0112	0.0148	NS	0.0332	0.0004	0.0004	NS	NS	< 0.0001	< 0.0001
When serious ADRs should be reported? Correct answer	43.12/44.23	PL	43.30*	28.98	19.10	7.62	41.38^c^	21.30	37.32	41.43	58.57	46.41&	11.52	20.09	21.98
		DE	39.90	35.12^a^	2.45	22.53	56.34^c^	31.90	11.76	66.06^f^	33.94	39.59^	29.31	9.12	21.98
		Pvalue	NS	NS	< 0.0001	< 0.0001	0.0010	0.0072	< 0.0001	< 0.0001	< 0.0001	0.0002	< 0.0001	< 0.0001	NS
**ATTITUDE**															
To whom should ADRs be reported? Correct answer	32.06/27.90	PL	38.46*	26.12	33.65	1.77	41.90^c^	39.36	18.74	41.18	58.82	37.55	31.96	12.87	17.62
		DE	28.87	33.32*	28.17	9.64	31.10	39.09	29.81	52.57	47.43	37.34^	31.92	1.76	28.98
		Pvalue	NS	NS	NS	0.0006	NS	NS	0.0214	0.0487	0.0487	NS	NS	0.0012	0.0163
Do you believe that many ADRs in children with epilepsy are preventable?: yes	34.10/38.88	PL	35.76*	31.12	30.43	2.69	44.10^c^	38.12	17.78	28.98^f^	71.02	29.41	42.88^	9.62	18.09
		DE	45.23*	16.38	27.15	11.24	29.09	38.65	32.26	29.47	70.53	33.32	26.15	15.43	21.09
		Pvalue	NS	< 0.0001	NS	< 0.0001	0.0035	NS	< 0.0001	NS	NS	0.0448	< 0.0001	0.0317	NS
Do you think it is necessary to report ADRs from children with epilepsy?: yes	37.79/40.32	PL	43.30*	28.98	19.10	7.62	41.38	21.30	37.32	41.43	58.57	56.41&^	9.52	12.09	21.98
		DE	39.90*	35.12	12.45	12.53	53.34	33.90	11.76	66.06^f^	33.94	39.59^	29.31	9.12	21.98
		Pvalue	NS	NS	0.0134	0.0204	NS	NS	< 0.0001	< 0.0001	< 0.0001	< 0.0001	< 0.0001	NS	NS
Do you think the ADRs reporting is a neurologist’s obligation?: yes	33.12/31.09	PL	40.15*	38.12	12.92	7.81	31.76	38.44	29.80	58.09	41.91	37.12^	29.04	15.12	18.72
		DE	40.09^a^	28.12	10.65	21.14	33.10	37.32	29.58	55.90	44.10	27.12#	31.87	33.76	7.25
		Pvalue	NS	NS	NS	0.0004	NS	NS	NS	NS	NS	NS	NS	0.0001	0.0036
**PRACTICE**															
Have you ever reported any ADRs from children with epilepsy?: yes	28.99/36.12	PL	42.40*	31.23	16.78	9.59	38.12	43.89^c^	17.99	41.12	58.88	43.09^	27.03	7.37	22.51
		DE	44.14*	26.76	18.46	10.64	43.76^c^	32.69	23.55	60.77^f^	39.23	52.08#	26.77	18.09	3.06
		Pvalue	NS	NS	NS	NS	NS	0.0302	NS	0.0002	0.0002	NS	NS	0.0012	< 0.0001
Do you report ADRs on a regular basis from children with epilepsy?: yes	23.34/28.12	PL	42.15*	38.12	11.92	7.81	31.76	38.44	29.80	58.09	41.91	37.12^	29.04	15.12	18.72
		DE	40.09^a^	28.12	10.65	21.14	33.10	37.32	29.58	55.90	44.10	27.12#	31.87	33.76	7.25
		Pvalue	NS	NS	NS	0.0004	NS	NS	NS	NS	NS	NS	NS	0.0001	0.0036
If yes, how many ADRs on average would be diagnosed (or observed) in a period of 6 months?															
< 5	53.20/57.09	PL	29.10	37.31*	18.87	14.72	44.09	29.68	26.23	65.90^f^	34.10	47.14^	26.05	5.72	21.09
		DE	44.09*	25.12	28.10	2.69	36.87	41.10^f^	22.03	53.50	46.50	39.21^	27.03	8.66	25.10
		Pvalue	< 0.0001	0.0002	0.0014	< 0.0001	0.0349	0.0005	NS	0.0003	0.0003	0.0216	NS	NS	NS
5–10	36.90/30.01	PL	56.41^ h^	9.52	12.09	21.98	51.11^d^	17.58	31.31	58.17	41.83	41.23&	12.38	27.15	19.24
		DE	39.59^a^	29.31	9.12	21.98	37.10	25.15	37.75	49.03	50.97	33.76#	29.12	33.43	3.69
		Pvalue	0.0368	0.0018	NS	NS	NS	NS	NS	NS	NS	NS	0.0102	NS	0.0027
> 10	9.90/12.90	PL	39.10*	35.43	17.85	7.62	39.76	30.44	29.80	47.43	37.34	29.97#	34.12	30.10	5.81
		DE	22.65	29.13	19.95	28.27	33.10	37.32	29.58	61.77^f^	38.23	40.09^	28.12	10.65	21.14
		Pvalue	NS	NS	NS	0.0310	NS	NS	NS	NS	NS	NS	NS	NS	NS
What type of ADRs is the one you report most frequently?															
severe	72.04/69.77	PL	37.55*	31.96	12.87	17.62	39.38	21.30	39.32	41.43	58.57	37.55^	29.00	14.14	19.31
		DE	37.34^a^	31.92	1.76	28.98	53.34^c^	33.90	11.76	66.06^f^	33.94	33.76#	30.12#	32.43#	3.69
		Pvalue	NS	NS	< 0.0001	0.0002	0.0002	0.0001	< 0.0001	< 0.0001	< 0.0001	NS	NS	< 0.0001	< 0.0001
rare	12.55/13.25	PL	6.64*	20.10	28.12	45.14	43.12^c^	37.89	18.99	48.12	51.88	56.41&	9.52	12.09	21.98
		DE	3.54*	28.90	31.90	35.66	48.76^c^	29.09	22.15	62.77^f^	37.23	39.59^	29.31	9.12	21.98
		Pvalue	NS	NS	NS	NS	NS	NS	NS	NS	NS	NS	NS	NS	NS
unexpected	15.41/16.98	PL	20.89	18.12	27.12	33.87	29.56	31.17	39.27	51.09	48.91	37.55^	31.96^	12.87	17.62
		DE	26.98	30.13	23.34	19.56	33.13	39.03	27.84	68.02^f^	31.98	37.34^	31.92^	1.76	28.98^
		Pvalue	NS	NS	NS	NS	NS	NS	NS	NS	NS	NS	NS	0.0415	NS

*PL *Poland, *DE *Germany, *NS *Not statistically significant difference (*p* > 0.05); * statistically significant difference (*p* < 0.05) vs. ˃50 y.o.a ; cstatistically significant difference (*p* < 0.05) vs. ˃20 years; ^ statistically significant difference (*p* < 0.05) vs. private practice/private office; a statistically significant difference (*p* < 0.05) vs. 41–50 y.o.a; d statistically significant difference (*p* < 0.05) vs. 11–20 years; f statistically significant difference (*p* < 0.05) vs. ≥ 20 average patient/day; g statistically significant difference (*p* < 0.05) vs. ≤ 30 y.o.a.; h statistically significant difference (*p* < 0.05) vs. 31–40 years; j statistically significant difference (*p* < 0.05) vs. 41–50 y.o.a; #statistically significant difference (*p* < 0.05) vs. other; & statistically significant difference (*p* < 0.05) vs. hospital; $ statistically significant difference (*p* < 0.05) vs. universities

43.12% pediatric neurologists from Poland and 44.23% form Germany knew when to report ADRs and 37.79% respondents form Poland and 40.32% from Germany felt co-responsible for reporting them, but relatively few neurologists knew where to report ADRs, especially among the German pediatric neurologists (Table 2). These results were correlated with sociodemographic data (*p* < 0.05) (Table 2).

Only 34.10% of pediatric neurologists from Poland, and 38.88% from Germany believe that many ADRs are preventable but almost most of the neurologists (PL-57.79%; DE – 45.32%) believe it is necessary to report ADRs from children with epilepsy. Interestingly, few pediatric neurologists believe that ADRs reporting is a neurologist’s obligation, it was observed especially among polish neurologists (Table 2).

Only 28.99% of the pediatric neurologists from Poland and 36.12% from Germany declared that they had reported ADRs at least once during their professional practice, and few of them (PL-23.34%3; DE-28.12%) declared regular reporting of such incidents. Most of the neurologists have reported only ˂5 ADRs in the last 6 months (PL-53.20%; DE-57.09%%), mostly the severe (PL-72.04%; DE- 69.77%) ADRs. It was correlated with sociodemographic data (p < 0.05), especially with age and years of experience (Table 2).

The most popular communication methods preferred by pediatric neurologists to send ADRs to an ADR reporting center were e-mail or website (PL-55.98%; DE- 61.01%), especially by the youngest neurologists with shorter years of experience. On the other hand, the oldest paediatrics neurologists preferred the traditional methods such as post office or direct contact as an option to report ADRs (Table 3).

**Table 3:** The most frequent methods used to report ADRs and sources used to gather information about ADRs.

**Which method would you prefer to send ADRs information to an ADR reporting center?**															
Email/on Website (%)	55.98/61.01	PL	42.15*	38.12	11.92	7.81	71.88^c^	25.00	3.12	41.35	58.65	37.55^	31.96^	12.87	17.62
		DE	40.09*	28.12	10.65	21.14	41.65	22.12	36.23	61.61^f^	38.39	37.34^	31.92^	1.76	28.98^
		Pvalue	NS	0.0300	NS	< 0.0001	< 0.0001	NS	< 0.0001	< 0.0001	< 0.0001	NS	NS	0.0001	0.0043
Direct contact (%)	19.36/10.77	PL	18.51	11.84*	29.98	39.67	5.76^c^	19.90	74.34	44.98	55.02	38.19^	31.39	4.30	26.12
		DE	11.58^a^	28.95	31.59	27.88	9.08^c^	27.77	63.15	61.04	38.96	29.90	36.15^	12.12	21.83
		Pvalue	NS	0.0042	NS	0.0549	NS	NS	NS	0.0155	0.0155	NS	NS	NS	NS
Telephone (%)	15.57/18.10	PL	10.90	10.12^j^	42.10	36.88	12.10^c^	36.39	51.51	72.09^f^	27.91	42.15#	38.12#	11.92	36.50$
		DE	12.17^a^	17.88	37.51	32.44	18.37^c^	29.03	52.60	66.43^f^	33.57	40.09^	28.12	10.65	22.96
		Pvalue	NS	NS	NS	NS	NS	NS	NS	NS	NS	NS	NS	NS	0.0118
Post (%)	9.09/10.12	PL	23.51	10.30^j^	37.13	29.06	18.98	27.13	53.89	65.17^f^	34.83	4.96	22.51	36.03$	36.50$
		DE	8.57*	25.43	27.88	38.12	21.03^c^	25.31	53.66	49.09	50.91	17.09	26.04	33.91	22.96
		Pvalue	0.0006	0.0006	NS	NS	NS	NS	NS	0.0053	0.0053	0.0006	NS	NS	0.0118
**The sources used to gather information about ADRs**:															
Textbooks (%)	9.12/13.09	PL	56.41^ h^	9.52	12.09	21.98	39.38	21.30	39.32	41.43	58.57	37.55	29.00	14.14	19.31
		DE	39.59*	29.31	9.12	21.98	53.34^c^	33.90	11.76	66.06^f^	33.94	33.76#	30.12#	32.43	3.69
		Pvalue	0.0047	< 0.0001	NS	NS	0.0185	0.0173	< 0.0001	< 0.0001	< 0.0001	NS	NS	0.0002	0.0001
Experience (%)	10.55/6.99	PL	9.93*	12.12	29.51	48.44	23.51	10.30^c^	66.19	56.88	43.12	12.17	17.88	37.51$	32.44$
		DE	19.74	18.06	25.66	36.54	8.57^c^	25.43	66.00	63.09^f^	36.91	10.90	10.12	42.10 $&	36.88 $&
		Pvalue	NS	NS	NS	0.0433	0.0013	0.0003	NS	NS	NS	NS	NS	NS	NS
Drug information sheets (%)	21.98/25.01	PL	42.15*	38.12	11.92	7.81	43.12^c^	37.89	18.99	48.12	51.88	56.41&	9.52	12.09	21.98
		DE	40.09^a^	28.12	10.65	21.14	48.76^c^	29.09	22.15	62.77^f^	37.23	39.59^	29.31	9.12	21.98
		Pvalue	NS	NS	NS	0.0017	NS	NS	NS	0.0217	0.0217	0.0086	< 0.0001	NS	NS
Journals (%)	11.94/11.11	PL	20.89	18.12	27.12	33.87	21.30	39.32	41.43	72.09	27.91	42.15#	38.12	11.92	7.81
		DE	26.98	30.13	23.34	19.56	33.90^c^	11.76	66.06	66.43^f^	33.57	40.09^	28.12^	10.65	21.14
		Pvalue	NS	NS	NS	NS	NS	0.0002	0.0036	NS	NS	NS	NS	NS	0.0271
Medical representatives (%)	10.01/8.12	PL	42.15*	38.12	11.92	7.81	71.88^c^	25.00	3.12	41.35	58.65	37.55^	31.96	12.87	17.62
		DE	40.09^ h^	28.12	10.65	21.14	41.65^d^	22.12	36.23	61.61	38.39	37.34^	31.92	2.76	28.98
		Pvalue	NS	NS	NS	NS	0.0161	NS	0.0018	NS	NS	NS	NS	NS	NS
Internet (%)	26.54/25.09	PL	42.01*	33.23	18.78	5.98	43.12^c^	37.89	18.99	48.12	51.88	43.09^	29.03	7.37	20.51
		DE	34.44^ h^	38.76	10.16	16.64	48.76^c^	29.09	22.15	62.77^f^	37.23	52.08#	26.77	18.09	3.06
		Pvalue	0.0240	NS	0.0005	< 0.0001	NS	0.0070	NS	< 0.0001	< 0.0001	0.0088	NS	< 0.0001	< 0.0001
Seminar/conferences (%)	7.12/5.99	PL	39.10*	35.43	17.85	7.62	39.76	30.44	29.80	47.43	37.34	29.97#	34.12#	30.10#	5.81
		DE	15.65*	23.13	16.55	38.27	33.10	37.32	29.58	61.77	38.23	40.09^	28.12	10.65	21.14
		Pvalue	0.0104	NS	NS	0.0007	NS	NS	NS	NS	NS	NS	NS	0.0180	0.0379
Drug promotional literature (%)	2.74/4.60	PL	12.41*	19.57	38.04	29.98	21.11^d^	47.58	31.31	58.17	41.83	41.23$	12.38	27.15	19.24
		DE	15.49*	21.31	29.12	34.08	37.10	25.15	37.75	49.03	50.97	33.76#	29.12	33.43	3.69
		Pvalue	NS	NS	0.0490	NS	0.0007	< 0.0001	NS	NS	NS	NS	0.0001	NS	< 0.0001

*PL *Poland, *DE *Germany, *NS *Not statistically significant difference (*p* > 0.05); * statistically significant difference (*p* < 0.05) vs. ˃50 y.o.a ; cstatistically significant difference (*p* < 0.05) vs. ˃20 years; ^ statistically significant difference (*p* < 0.05) vs. private practice/private office; a statistically significant difference (*p* < 0.05) vs. 41–50 y.o.a; d statistically significant difference (*p* < 0.05) vs. 11–20 years; f statistically significant difference (*p* < 0.05) vs. ≥ 20 average patient/day; g statistically significant difference (*p* < 0.05) vs. ≤ 30 y.o.a.; h statistically significant difference (*p* < 0.05) vs. 31–40 years; j statistically significant difference (*p* < 0.05) vs. 41–50 y.o.a; #statistically significant difference (*p* < 0.05) vs. other; & statistically significant difference (*p* < 0.05) vs. hospital; $ statistically significant difference (*p* < 0.05) vs. universities

The main sources used to gather information about ADRs by the Polish pediatric neurologists included the Internet (78.09%) and drug information sheets experience (21.98%). Among the German pediatric neurologists, the most popular sources of information about ADRs were the Internet (82.12%) and journals (25.01%) (Table 3).

The main reason for the pediatric neurologists not to report ADRs was the concern that the report will generate extra work(PL- 27.77%; DE- 23.06%) and a poor level of knowledge which makes it difficult to decide whether or not an ADRs has occurred ( PL- 24.00%; DE- 26.98%). It was correlated with sociodemographic data (p < 0.05) (Table 4). The largest percent of the Polish pediatric neurologists (25.54%) claim that the institutional role should be more active to improve the PV system in practice. In turn, in the opinion of German pediatric neurologists’, the urgent activities that should be implemented into practice is “ADRs reporting should be compulsory in-service training”. Both, Polish and German neurologists are in favor of including a PV exercise in undergraduate examination (PL-28.99%; DE-27.78%) (Table 4). 16% of pediatric neurologists from Poland and 18.62% from Germany also indicated “Strengthen training program on ADR reporting” as other activities which should be implemented into the health system to improve PV (Table 4).

**Table 4:** The factors which may discourage pediatric neurologists from delivering pharmacovigilance and activities to improve spontaneous ADRs reporting (*n* = 1095)

	Response N(%) PL/DE	Country	Age (%) PL/DE				Years of experience(%) PL/DE			Average patient with epilepsy per day(%) PL/DE		Place of employment(%) PL/DE			
			≤ 30	31–40	41–50	>50	≤ 10	11–20	>20	< 20	≥ 20	Universities	hospital	private practice/private office	other
**BARRIERS**															
Apprehension about sending in an inappropriate report	1.35/2.00	PL	39.10*	35.43	17.85	7.62	39.76	30.44	29.80	47.43	37.34	29.97#	34.12#	30.10#	5.81
		DE	15.65	23.13	22.95	38.27^ g^	33.10	37.32	29.58	61.77^f^	38.23	40.09^	28.12	10.65	21.14
		Pvalue	< 0.0001	0.0327	NS	< 0.0001	NS	NS	NS	0.0218	NS	NS	NS	0.0002	0.0002
Lack of time to fill in a report	18.05/16.12	PL	9.93*	12.12	29.51	48.44	23.51	10.30^c^	66.19	56.88	43.12	12.17	17.88	37.51$	32.44$
		DE	19.74	18.06	25.66	36.54	8.57^c^	25.43	66.00	63.09^f^	36.91	10.90	10.12	42.10 $&	36.88 $&
		Pvalue	NS	NS	NS	NS	0.0005	< 0.0001	NS	NS	NS	NS	< 0.0001	NS	< 0.0001
Concern that the report will generate extra work	27.77/23.06	PL	42.01*	33.23	18.78	5.98	43.12^c^	37.89	18.99	48.12	51.88	43.09^	29.03	7.37	20.51
		DE	34.44^ h^	38.76	10.16	16.64	48.76^c^	29.09	22.15	62.77^f^	37.23	52.08#	26.77	18.09	3.06
		Pvalue	0.0240	NS	0.0005	< 0.0001	NS	0.0070	NS	< 0.0001	< 0.0001	0.0088	NS	< 0.0001	< 0.0001
Absence of a fee for reporting ADRs	14.07/16.55	PL	46.09*	34.20	11.90	7.81	71.88**	25.00	3.12	41.35	58.65	37.55^	31.96	12.87	17.62
		DE	40.09^a^	28.12	10.65	21.14	41.65^d^	22.12	36.23	61.61^f^	38.39	37.34^	31.92^	1.76	28.98^
		Pvalue	NS	NS	NS	0.0208	0.0002	NS	< 0.0001	0.0151	0.0151	NS	NS	0.0131	NS
Level of knowledge makes it difficult to decide whether or not an ADRs has occurred	24.00/26.98	PL	40.15*	38.12	12.92	7.81	31.76	38.44	29.80	58.09	41.91	37.12^	29.04	15.12	18.72
		DE	40.09^a^	28.12	10.65	21.14	33.10	37.32	29.58	55.90	44.10	27.12#	31.87	33.76	7.25
		Pvalue	NS	NS	NS	0.0004	NS	NS	NS	NS	NS	NS	NS	0.0001	0.0036
Do not feel the need to report reactions reported by patients	8.67/9.12	PL	12.41	19.57	38.04^ g^	29.98	21.11^**^	31.31	47.58	58.17	41.83	27.15	12.38	41.23&	19.24
		DE	15.49	21.31	29.12	34.08^ g^	37.10	25.15	37.75	49.03	50.97	3.69	29.12$	33.43 $	33.76$
		Pvalue	NS	NS	NS	NS	NS	NS	NS	NS	NS	NS	NS	NS	NS
Physicians’ yellow cards not available when needed	6.09/6.17	PL	39.10*	35.43	17.85	7.62	39.76	30.44	29.80	47.43	37.34	29.97#	34.12#	30.10#	5.81
		DE	15.65	23.13	22.95	38.27^ g^	33.10	37.32	29.58	61.77^f^	38.23	40.09^	28.12	10.65	21.14
		Pvalue	< 0.0001	0.0327	NS	< 0.0001	NS	NS	NS	0.0218	NS	NS	NS	0.0002	0.0002
**ACTIVITIES**															
Strengthen training program on ADR reporting	16.12/18.62	PL	40.15*	38.12	12.92	7.81	31.76	38.44	29.80	58.09	41.91	37.12^	29.04	15.12	18.72
		DE	40.09^a^	28.12	10.65	21.14	33.10	37.32	29.58	55.90	44.10	27.12#	31.87	33.76	7.25
		Pvalue	NS	NS	NS	0.0004	NS	NS	NS	NS	NS	NS	NS	0.0001	0.0036
ADRs reporting should be compulsory in-service training	13.34/22.88	PL	36.41^a^	29.52	12.09	21.98	39.38	21.30	39.32	41.43	58.57	33.55	25.00	22.14	19.31
		DE	39.59^a^	29.31	9.12	21.98	53.34**	33.90	11.76	66.06^f^	33.94	33.76#	30.12#	32.43#	3.69
		Pvalue	NS	NS	NS	NS	0.0318	0.0261	< 0.0001	0.0002	0.0002	NS	NS	NS	0.0005
Institutional role should be more active	25.54/15.26	PL	14.77	15.88	51.45^ g^	17.09	10.87**	32.15	56.98	73.98^f^	26.02	44.98^	31.09^	3.49	20.44^
		DE	14.50	13.46	29.04	43.00^ g^	12.79**	39.90	47.31	57.22	42.78	38.86^	29.49	13.60	18.05
		Pvalue	NS	NS	< 0.0001	< 0.0001	NS	NS	NS	0.0009	0.0009	NS	NS	0.0003	0.5765
Report forms should be included in prescribing pad	9.03/11.66	PL	9.93	12.12	29.51	48.44^ g^	23.51	10.30	66.19^d^	56.88	43.12	12.17	17.88	37.51$	32.44
		DE	19.74	18.06	25.66	36.54^ h^	8.57**	25.43	66.00	63.09	36.91	10.90	10.12	42.10 $&	36.88 &$
		Pvalue	NS	NS	NS	NS	0.0401	0.0538	NS	NS	NS	NS	NS	NS	NS
An uncomplicated reporting system with quick feedback	6.98/3.8	PL	39.10*	35.43	17.85	7.62	39.76	30.44	29.80	47.43	37.34	29.97#	34.12#	30.10#	5.81
		DE	15.65*	23.13	16.55	38.27	33.10	37.32	29.58	61.77	38.23	40.09^	28.12	10.65	21.14
		Pvalue	0.0104	NS	NS	0.0007	NS	NS	NS	NS	NS	NS	NS	0.0180	0.0379
Exercise should be included in undergraduate examination	28.99/27.78	PL	20.89	18.12	27.12	33.87	21.30	39.32	41.43	72.09	27.91	42.15#	38.12	11.92	7.81
		DE	26.98	30.13	23.34	19.56	33.90^c^	11.76	66.06	66.43^f^	33.57	40.09^	28.12^	10.65	21.14
		Pvalue	NS	NS	NS	NS	NS	0.0002	0.0036	NS	NS	NS	NS	NS	0.0271

PL- Poland; DE-Germany; NS- not statistically significant difference (p > 0.05); * statistically significant difference (p < 0.05) vs. ˃50 y.o.a ; cstatistically significant difference (p < 0.05) vs. ˃20 years; ^ statistically significant difference (p < 0.05) vs. private practice/private office; a statistically significant difference (p < 0.05) vs. 41–50 y.o.a; d statistically significant difference (p < 0.05) vs. 11–20 years; f statistically significant difference (p < 0.05) vs. ≥ 20 average patient/day; g statistically significant difference (p < 0.05) vs. ≤ 30 y.o.a.; h statistically significant difference (p < 0.05) vs. 31–40 y.o.a.; j statistically significant difference (p < 0.05) vs. 41–50 y.o.a; #statistically significant difference (p < 0.05) vs. other; & statistically significant difference (p < 0.05) vs. hospital; $ statistically significant difference (p < 0.05) vs. universities; @ statistically significant difference (p < 0.05) vs. ≤ 10 years;** statistically significant difference (p < 0.05) vs. ˃20 years

## Discussion

Based on the lack of literature concerning a similar theme of the study, it seems believed that it is an innovative study. Besides, to strengthen the value of the study, the results were gathered from two countries- Poland and Germany, and comparison analysis was performed. The presented study is a continuation of a larger study [11] whose aim was to assess whether the effect of a “well-established PV position” in the existing healthcare system influences compliance with the obligation to participate in national PV and ADR reporting by pediatric neurologists.

Due to the lack of literature based on pediatric neurologics as a study group, the discussion refers to studies involved general professional group, i.e. medical doctors.

The results of the study have shown that the pediatric neurologists had a good knowledge of the general issues connected with PV process and such knowledge was dependent on the age of the respondents. It was similar to previous observations [12, 13]. Despite having such a good knowledge of PV and ADR, the neurologists participating in our study were unaware of the existence of pharmacotherapy safety monitoring centers responsible for PV in their countries [14]. Similar conclusions were reached by researchers from Malaysia, who confirmed that this was the main reason (40%) for not reporting ADRs [15–18]. One way to address this problem is to incorporate PV as an essential part of healthcare personnel training, especially among doctors. The vast majority of pediatric neurologists did not recognize the contribution of other healthcare professionals as potential ADR reporters [19, 20], but they were aware of their responsibility to include PV in their daily duties, including PV reporting as part of their daily responsibilities. For instance, in one of the recent german study [14] 54% of responders said that ADRs play a minor role in their routine care, and 4 (3%) stated that they play no role at all.

In addition, most neurologists knew the timeframe for reporting major ADRs, but this was not correlated with the number of reported ADRs seen in children with epilepsy, as the vast majority reported that they only reported < 5 ADRs in the last 6 months. This may be due to a number of systemic factors indicated by both Polish and German neurologists as barriers to PV processing. Among the barriers in carrying out PV, the pediatric neurologists participating in our study most often mentioned the fear that the report will generate additional work and poor lack of knowledge about general PV process. It was similar to other studies [19, 21, 22], in which the main reason for underreporting ADRs was lack of time, little knowledge of the types of reactions to be preferentially reported and also the absence of a fee for reporting ADRs.

The results of the study suggested a positive attitude of pediatric neurologists to the PV process, which is very positive, as was observed in other studies in which participants were eager to learn and apply the knowledge about ADR reporting in their daily routine [17]. It was similar among the pediatric neurologists from Poland and Germany, as well. Unfortunately, besides such a positive attitude toward general PV practice, a small number of pediatric neurologists believed that ADR reporting was their professional obligation, especially among polish pediatric neurologists’ (33.12%). It is naturally correlated with the next statements among polish neurologists concerning the regular basis of ADRs reporting- only 23.34% of polish pediatric neurologists’ declared ADRs reporting on their a regular basis. The same small percentage in mentioned fields was presented by German pediatric neurologists, so we can assume that both compared countries have the same problem with a legal obligation to participate in the national and European PV process. A significant percentage of neurologists also believed that only serious adverse events should be considered important or were unsure about the types of adverse events that should be reported. This finding was consistent with previous studies [18, 19]. It is important to acknowledge that less severe and atypical adverse events are also significant, as they can serve as indicators of the potential occurrence of fatal adverse events in the future. Factors identified by physicians as barriers to reporting adverse events should be promptly addressed, including the barriers mentioned earlier [20].

We also inquired about the frequency of reporting ADRs and the number of reported ADRs within a 6-month period. The most common response from pediatric neurologists was “yes” regarding regular reporting, while the most common response to the second question was “<5.“ According to various research findings, physicians’ practice in reporting ADRs fell significantly below expectations. Meanwhile, the pace of reporting ADRs to the appropriate regulatory bodies was quite overwhelming, with a majority of physicians who encountered ADRs submitting few reports or not reporting at all [21]. Surveys done in Malaysia have shown that only 5.3% of doctors had ever reported ADRs [21], a similar result was found in UAE 11% [22].

Similarly, a study conducted in Romania found that 79.9% of surveyed doctors did not report any ADRs [23], and a comparable result was obtained in India 77% [24]. In contrast, an article from Sweden yielded a positive outcome, with 62% of doctors having ever reported an ADR [18].

The research findings indicate the most commonly used sources of obtaining information about adverse drug reactions (ADRs). The most popular source of information among neurologists in Poland was the Internet and medication package inserts, while in Germany, it was journals and the Internet. Studies conducted in Pakistan revealed that 24% of doctors refer to the Internet, 33.6% to seminars, 18.4% to journals, and 10.4% to drug advert [25], similarly, in Nigeria 41.4% refer to books/ journals, 18.3 to seminars/ training, 4.4% to the internet [26], and in India 63% of doctors identified the internet as the source of information, 65% seminars, 69% journals, 40% medical books [24], other doctors (89%) emphasised the role of information technology [19], 93.6% [27], and 75% [28].

The pediatric neurologists in our study suggested different activities which should be implemented for the improvement of the PV process, among many of these activities the most frequently mentioned were strengthening the training program on ADR reporting, activating institutional role in ADRs reporting, and also including reporting exercise in the undergraduate examination as an important tool for increasing physicians’ awareness of ADRs in practice. In 2009, Oshikoya and Awobusuyi also recommended including pharmacovigilance as a topic in continuing education programmes [29]. Various studies have shown that the optimization of the knowledge, attitude, and practices about pharmacovigilance is essential to promote reporting [30, 31].

The participants also encouraged the governments to take the necessary steps to ensure the safe and effective use of drugs among the population. In addition, pediatric neurologists to improve the PV system, including lifelong learning, seminars as well as training. The literature also confirms that ensuring optimal knowledge, awareness of attitudes and PV practices is essential to promoting ADR reporting [30, 31]. Globally, in the developed world, ADR reporting is shifting from the prescribing physician to the consumer or patient.

## Conclusion

To conclude, monitoring the safety of pharmacotherapy and knowledge of risks associated with ADRs should be included in the curricula of academic physicians’ courses. Pediatricn eurologists from Poland and Germany have good knowledge of PV and ADRs reporting. Both, the Polish and German pediatric neurologists demonstrated a positive attitude toward ADRs reporting and understood the importance of PV in the general concept of ensuring pharmacotherapy safety for children with epilepsy. However, it seems that technical ability and a good attitude to provide PV is insufficient to include PV in practice. There is a huge need to improve the general system of PV both in Germany and Poland and encourage pediatric neurologists to regular PV on daily routine work.

## Supplementary Information

**Additional file 1.** Supplementary materials questionnaire.

## Data Availability

The datasets used and/or analysed during the current study available from the corresponding author on reasonable request.
