# Supplementary material for: Pharmacovigilance practice among pediatric neurologists from Poland and Germany
Source: BMC Med Educ. 2023 Aug 1;23:547. doi: 10.1186/s12909-023-04542-4 (PMC10394771; doi:10.1186/s12909-023-04542-4)
Supplement: Supplementary file 1 — Additional file 1. Supplementary materials questionnaire. [file 12909_2023_4542_MOESM1_ESM.docx]

**QUESTIONNAIRE**

**(PHARMACOVIGILANCE AMONG PHYSICIANS)**

**CHARACTERISTICS OF PHYSICIANS**

**Do you agree to participate in this study after reading information about it (informed consent)?**

**a. yes**

**b. no**

**Gender**

a. Male b. FemaleP

**Age (years)**

a. ≤30 b. 31- 40 c. 41- 50 d. ˃50

**Years of experience**

a. ≤10 b. 11-20 c. ˃20

**Place of employment**

a. universities b. state hospital c. private hospital d. other (family, public,

occupational, or institutional health centers, private physicians’ office, and pharmaceutical company)

**Average patient with epilapsy per day:**

a. <20 b. ≥20

**KNOWLEDGE**

**1. The most appropriate definition of pharmacovigilance is?**

a. The science of monitoring adverse drug reactions (ADRs) happening in a hospital

b. The process of improving the safety of drugs

c. The detection, assessment, understanding and prevention of adverse effects

d. The science of detecting the type and incidence of ADR after a drug is marketed

**2. The most appropriate purpose of pharmacovigilance is?**

1. To identify the safety of drugs

2. To calculate the incidence of ADRs

3. To identify predisposing factors of ADRs

4. To identify unrecognized ADRs

**4. Functions of pharmacovigilance include the following, please indicate:**

a. Drug related problems

b. Herbal product

c. Medical devices and vaccines

d. All of the above

**5. ADR can be described as:**

a. Untoward medical occurrence that may be present during the treatment

b. Exaggeration of desired therapeutic effect which is usually not common at normal dose

c. Response to a drug which is noxious and unintended and occurs at dose normally used in man for prophylaxis, diagnosis, or treatment

d. Response to a drug which occur via different mechanism from the main action of the drug

**5. What is the purpose of an adverse drug reaction?**

a. Identify safe drugs

b. Measure the incidence of ADRs

c. Identify predisposing factors to ADRs

d. Identify new ADRs

e. Comparison of ADRs within the same class

**6. When serious ADRs should be reported?**

a. One day

b. Seven calendar days

c. Fifteen calendar days

d. Fourteen calendar days

**8. To whom should ADRs be reported?**

a. Ministry of Health

a. National Health Found

c. Office for Registration of Medicinal Products, Medical Devices and Biocidal Products

**10. Are you coresponsible for ADRs reporting?**

a. yes

b. no

**12. Do you believe that many ADRs are preventable?**

a. yes

b. no

**ATTITUDE**

**1. Do you think it is necessary to report ADRs from pediatric patients with epilepsy?**

a. yes

b. no

**3. Do you think the ADRs reporting is a pediatric neurologist’s obligation?**

a. yes

b. no

**PRACTICE**

**1. Have you ever reported any ADRs from pediatric patient with epilepsy?**

a. yes

b. no

**2. Do you report ADRs on a regular basis from pediatric patients with epilepsy?**

a. yes

b. no

**3. If yes, how many ADRs on average would be diagnosed (or observed) in a period of 6 months?**

a. <5

b. 5–10

c. >10

**4. What type of ADRs is the one you report most frequently?**

a. Severe

b. rare

c. unexpected

**5. Please indicate the most frequent AE you reported from pediatric patient with epilepsy?**

a. Irritability

b. Dizziness

c. Memory impairement

d. Dispiritedness

e. Oversleeping

f. Anxiety

g. Headache

h. Diplopia

i. Lack of libido

j. Gingival bleeding or hypertropy

k. Fatique

k. other (please specify)…………………………

**5. Which method would you prefer to send ADRs information to an ADR reporting center?**

a. Email/on Website

b. Direct contact

c. Telephone

d. Post

e. Other (e.g. mobile application)

**6. Please, indicate the sources used to gather information about ADRs (multiple choice possible):**

a. Textbooks

b. Experience

c. Drug information sheets (in drug packs)

d. Journals

e. Medical representatives

f. Internet

g. Seminar/conferences

h. Drug promotional literature

**BARRIERS**

**Please, indicate the factors which may discourage pediatric neurologists from delivering pharmacovigilance.**

a. Apprehension about sending in an inappropriate report

b. Lack of time to fill in a report

c. Concern that the report will generate extra work

d. Absence of a fee for reporting ADRs

e. Level of knowledge makes it difficult to decide whether or not an ADRs has occurred

f. Do not feel the need to report reactions reported by patients

g. Physicians' yellow cards not available when needed

**ACTIVITIES TO IMPROVE SPONTANEOUS ADRs REPORTING**

a. Strengthen training program on ADR reporting

b. ADRs reporting should be compulsory in-service training

c. Institutional role should be more active

d. Report forms should be included in prescribing pad

e. An uncomplicated reporting system with quick feedback

f. Reporting exercise should be included in undergraduate examination
